# Hip pain in childhood

**DOI:** 10.1590/0100-3984.2018.0042

**Published:** 2020

**Authors:** Sariane Coelho Ribeiro, Kaline Silva Santos Barreto, Catarina Borges Santana Alves, Oswaldo Lima Almendra Neto, Marcel Vieira da Nóbrega, Leonardo Robert de Carvalho Braga

**Affiliations:** 1 UDI 24 horas, Teresina, PI, Brazil.; 2 Universidade Ceuma, São Luís, MA, Brazil.; 3 Hospital São Carlos, Fortaleza, CE, Brazil.

**Keywords:** Hip joint, Pain/etiology, Arthritis, juvenile, Hip dislocation, congenital, Child, preschool, Child, Adolescent

## Abstract

Hip pain in a child can have infectious, inflammatory, traumatic, neoplastic, or developmental causes, which can make the diagnosis challenging. Meticulous history taking and a detailed clinical examination guide the radiological investigation. In this article, we address some of the main causes of hip pain in childhood and their findings on diagnostic imaging.

## INTRODUCTION

Hip pain in a child is a common reason for medical consultations and often represents a diagnostic challenge, because it can have numerous causes^(1)^, as detailed in Table 1.

**Table 1:** Causes of isolated hip pain in children.

Type		Disorder
Congenital*		Hip dysplasia
Infectious		Osteomyelitis^‡^
		Pyomyositis
		Septic arthritis*
Inflammatory		Transient synovitis of the hip*
		'Juvenile inflammatory arthritis
Traumatic/mechanical^†^		Epiphysiolysis
		Apophysitis
		Stress fracture
Neoplastic	Benign^†^	Osteoid osteoma
		Osteochondroma
		Eosinophilic granuloma
		Unicameral bone cyst
		Aneurysmal bone cyst
	Malignant	Metastases^‡^
		Ewing sarcoma^†^
		Leukemia, lymphoma^†^

* Typically present in children under 10 years of age.

† Typically present in older children or adolescents (≥ 10 years of age).

‡ Present in children of any age.

A number of studies recently conducted in Brazil have underscored the important role that imaging methods play in the evaluation of the musculoskeletal system^(2-7)^. Accurate diagnosis of pediatric hip disorders is essential for the characterization of early musculoskeletal changes^(8)^. The diagnostic imaging methods employed in the evaluation of hip pain are characterized in Table 2.

**Table 2:** Imaging methods for the diagnosis of hip pain in children.

X-ray	Initial method for evaluation of bone diseases
Ultrasound	Complementary to X-ray for better assessment of soft tissues and detection of joint effusion; useful for guiding punctures
Magnetic resonance imaging (MRI)	Evaluation of complications, prolonged complaints, and extent of disease; better joint detailing; and evaluation of the physeal cartilage, subchondral bone, periosteum, synovia, and bone marrow
Computed tomography (CT)	When there is suspicion of osteoid osteoma and for evaluation of the bone contours
Bone scintigraphy	When there is suspicion of osteomyelitis and the primary site is unknown; for the assessment of multifocal disease; and for detection of osseous lesions caused by stress/failure

## DISCUSSION

The common causes of hip pain in childhood include congenital disorders and developmental dysplasia, as well as infectious, inflammatory, traumatic, rheumatologic, and neoplastic processes^(1)^.

### Congenital disorders

#### Hip dysplasia

Hip dysplasia is characterized as an abnormal ratio between the femoral head and the acetabulum^(9)^. Suspicion of the condition usually arises during the first 30 days of life. For infants ≤ 6 months of age, Graf’s method of ultrasound exam can be used for screening and confirmation, thus allowing early preventive treatment to be provided^(9)^. 

**Figure 1:**
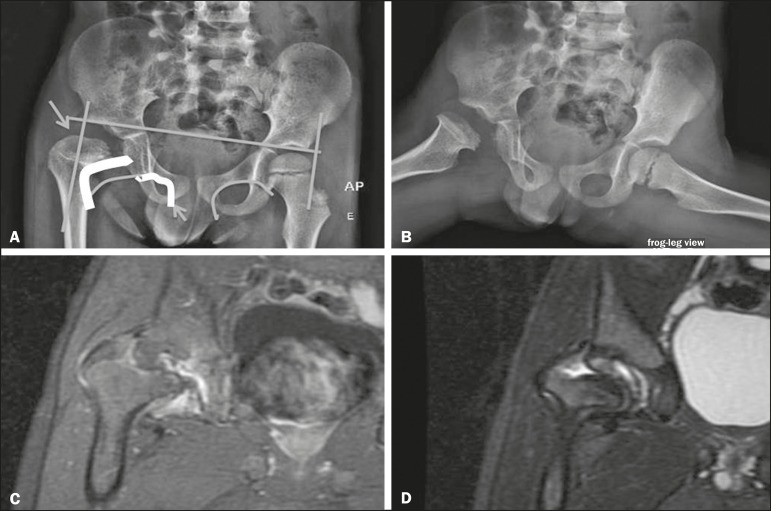
Dysplasia of the right hip in a 4 year-old male. Anteroposterior and frog-leg view X-rays (**A** and **B**, respectively), showing asymmetry of the hip joints, with right hip dysplasia. MRI of the right hip. Gadolinium-enhanced coronal, fat-suppressed T1-weighted sequence (**C**) and STIR sequence (**D**) showing a loss of sphericity, flattening, and superolateral subluxation of the right femoral head, with enlargement of the femoral neck and acetabular cavity, as well as joint effusion with signs of reactive synovitis.

On X-rays of infants with suspected hip dysplasia, we should look for symmetry of the hips by tracing the Hilgenreiner, Perkin and Shenton lines, as well as measuring the acetabular angle (Figure 1). Findings on X-rays suggest established dysplasia^(10)^. CT and MRI of the hip are useful in treatment planning, monitoring, and postoperative evaluation (Figure 1).

**Figure 2:**
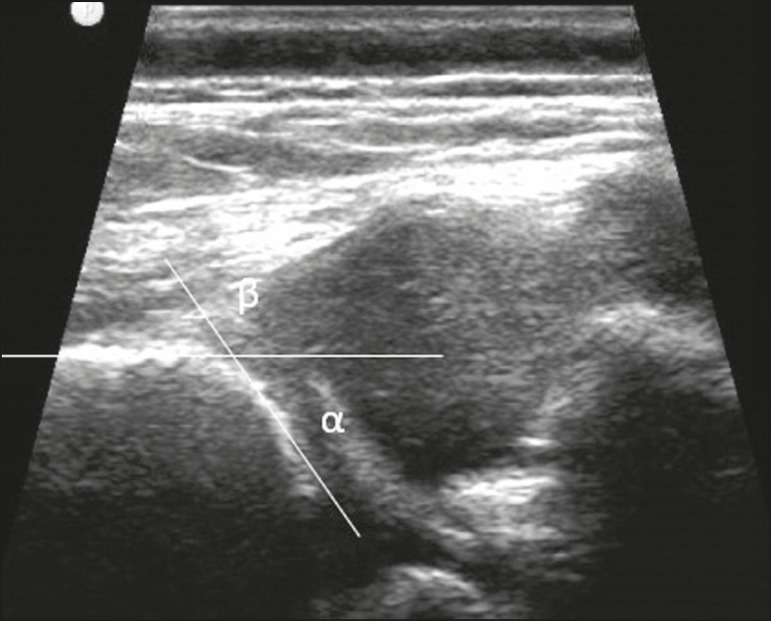
A 40-day-old child presenting left hip dysplasia, characterized by a alpha angle < 60º.

If left untreated, hip dysplasia can evolve to abnormalities of gait, discrepancies in the length of the limbs, early osteoarthritis, and avascular necrosis^(9)^ (Figure 2).

### Infectious processes

Among the infectious diseases that can affect children are osteomyelitis, pyomyositis, and septic arthritis. The most common infectious agents are *Staphylococcus aureus* in children and *Streptococcus agalactiae* in neonates. These pathogens spread either by hematogenous dissemination or by direct or indirect contamination^(1)^.

The clinical presentation of infectious diseases in children includes pain, local edema, fever with biochemical changes (increases in the erythrocyte sedimentation rate and C-reactive protein level), positive culture, and, in the case of pyomyositis, increased creatine phosphokinase and myoglobinuria^(1,10)^.

#### Osteomyelitis/septic arthritis

Osteomyelitis and septic arthritis often occur in the metaphyseal region of the bone marrow. Osteomyelitis typically affects males between 2 and 12 years of age, whereas septic arthritis affects children of either gender between 4 and 16 years of age^(10)^. 

**Figure 3:**
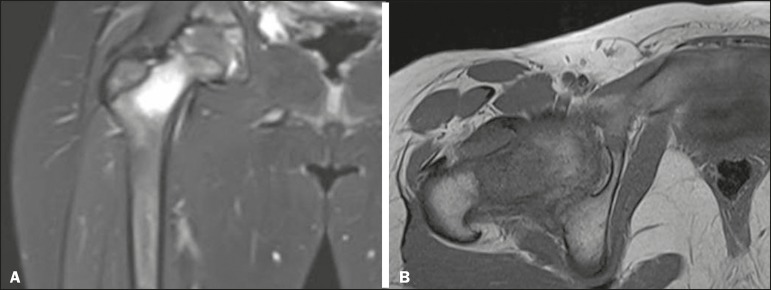
Osteomyelitis in a 9-year-old female. MRI of the right thigh. Coronal fat-suppressed T2-weighted sequence (**A**) and axial T1-weighted sequence (**B**) identifying an area in the marrow of the right femoral neck and extending to adjacent muscle planes, with a hypointense signal on the T1-weighted sequence, a hyperintense signal on the T2-weighted sequence, a mild periosteal reaction, and no erosion of cortical bone.

In osteomyelitis, X-rays and ultrasound are poorly sensitive in the early stages (within the first 10 days). Scintigraphy has high sensitivity but low specificity, being useful for verifying multifocality and for a diagnosis of exclusion when negative^(10)^. Bone destruction, cortical erosion, and periosteal reaction are all better visualized on CT scans. In the absence of trauma, a fat-fluid level is suggestive of osteomyelitis^(10)^. MRI is quite effective in the early diagnosis of osteomyelitis, as well as in assessing the extent of bone, soft tissue, and articular involvement. Osteomyelitic lesions show a hypointense signal on T1-weighted sequences and a hyperintense signal on fluid-sensitive sequences. In the post-contrast phase (Figure 3), MRI detects collections in soft, subperiosteal, and intraosseous tissues, with marginal contrast enhancement, as well as areas of osteonecrosis and fistulous tracts^(10)^.

#### Pyomyositis

Pyomyositis is a bacterial infection of the muscle. It typically affects children 5-9 years of age and is more common in males^(11)^.

**Figure 4:**
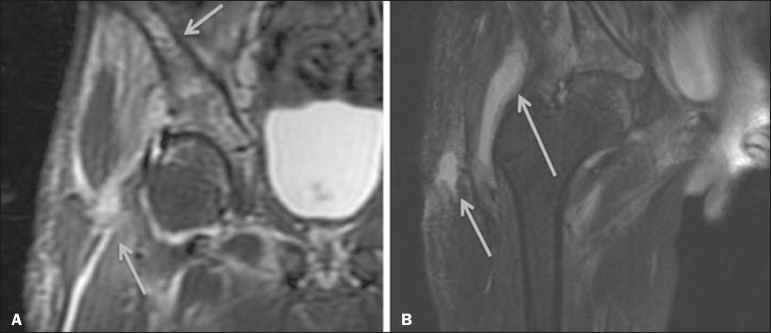
Pyomyositis in a 12-year-old male patient. Coronal fat-saturated T2-weighted MRI sequence showing joint effusion, osteitis, and edema involving the muscle-adipose planes of the gluteal region and root of the right thigh (**A**), featuring a voluminous, heterogeneous liquid collection, containing small foci of low signal intensity, likely of an infectious/inflammatory nature, located between the ventral portion of the gluteus medius muscle and that of the gluteus maximus muscle (**B**).

Ultrasound is useful in detecting muscle abscesses, although the findings can be unremarkable in the early stages of pyomyositis and osteomyelitis^(11)^. CT is not sufficiently sensitive to evaluate soft tissues or to differentiate between abscesses and hematomas^(11)^. MRI is the best examination for early diagnosis of soft-tissue disorders, as well as for concomitant arthritis or osteomyelitis, upon evaluation of contrast-enhanced fluid-sensitive sequences (Figure 4)^(11)^. 

**Figure 5:**
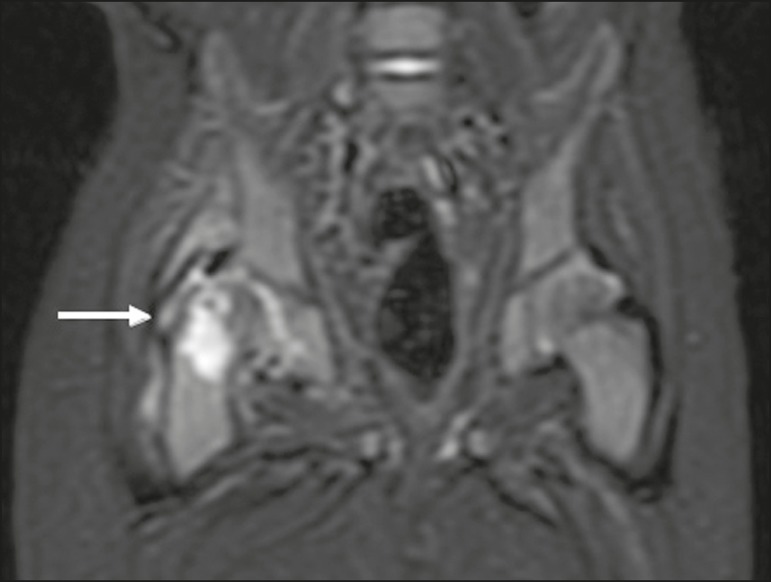
MRI of the hip. STIR coronal sequence showing mild effusion in the right hip joint, with synovial thickening and hyperintense signal/edema in the adjacent planes of the capsule of the muscle. Note the irregular lesion, with a hyperintense signal in a T2-weighted sequence, in the right femoral head and neck, which is suggestive of a focus of osteomyelitis/intraosseous abscess.

The X-ray findings in infectious arthritis include joint effusion, periarticular osteoporosis, reduction of the joint space, bone erosion, bone destruction, and, occasionally, ankylosis (late changes) and radiolucent foci, together with periosteal reactions or heterotopic bone formations^(10)^. Ultrasound is useful for guiding aspiration biopsy and detecting joint effusion. Scintigraphy is useful for determining the location and distribution of infectious processes^(10)^. As illustrated in Figure 5, MRI facilitates the detection of synovitis, inflammatory changes, and periarticular collections^(10)^.

The differential diagnosis of septic arthritis primarily includes transient synovitis of the hip and arthritis caused by rheumatoid or hematological diseases.

### Inflammatory processes

#### Transient synovitis

Transient synovitis of the hip is a benign condition, of unknown origin, that is self-limited. It accounts for approximately 90% of cases of hip pain in children. The majority of cases are in individuals with a history of upper respiratory tract infection (approximately two to three weeks before the onset of symptoms) or mild trauma. The age at onset ranges from 18 months to 13 years, most cases occurring in individuals between 3 and 8 years of age^(8)^.

**Figure 6:**
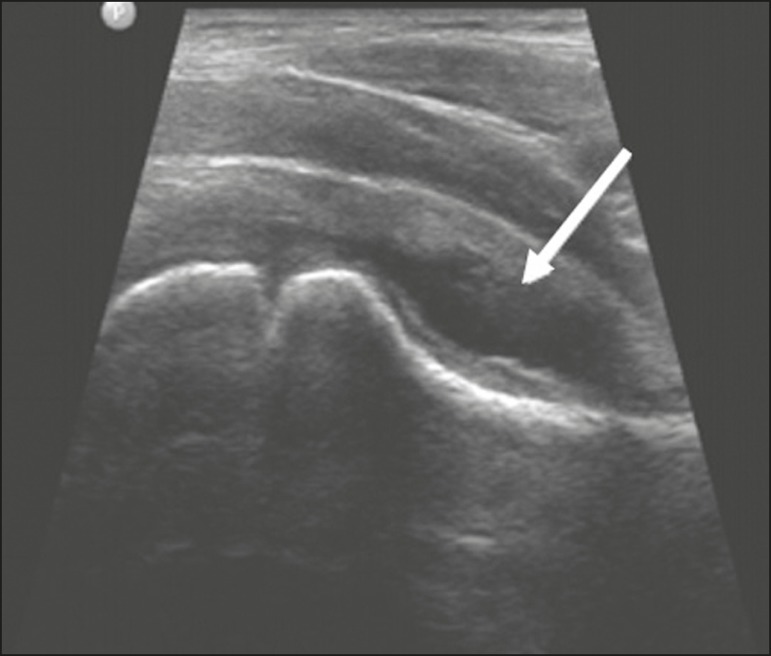
Ultrasound of the hip identifying joint effusion.

**Figure 7:**
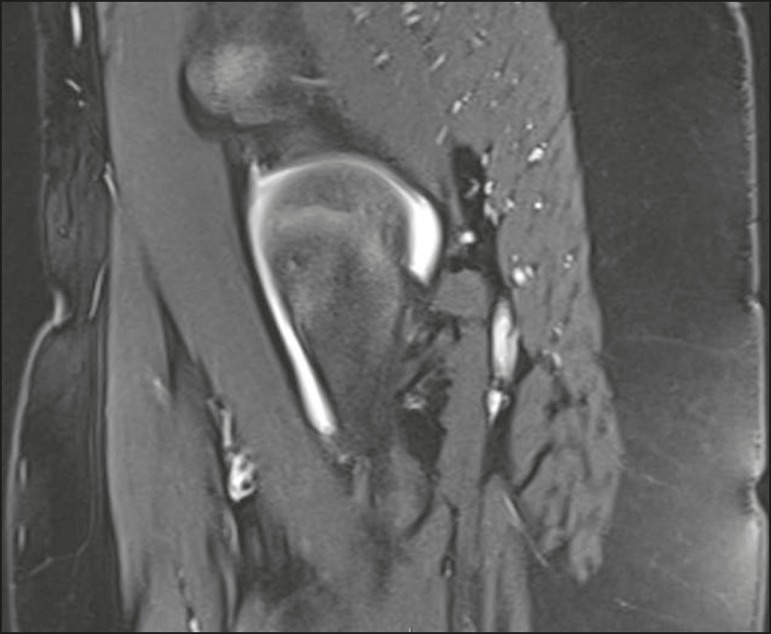
A 12-year-old male patient. Sagittal fat-saturated T2-weighted MRI sequence showing moderate intra-articular effusion in the right hip joint.

The diagnosis is made by exclusion. Ultrasound reveals anechoic joint effusion or with fine debris (Figure 6). An X-ray can show enlargement of the joint space and helps exclude other disorders of the hip. Other imaging exams are usually unnecessary. In cases in which there is suspicion of an infectious process, joint puncture is indicated^(8)^. The MRI findings include unilateral or bilateral joint effusion, as well as mild synovial thickening and enhancement. As can be seen in Figure 7, there is usually no clear alteration of the bone signal^(12)^.

### Trauma

#### Epiphysiolysis

Epiphysiolysis is best described as a fracture of the growth plate with a slipped capital femoral epiphysis (equivalent to a Salter-Harris type I fracture). It is the most common abnormality of the hip in overweight male adolescents^(10)^.

**Figure 8:**
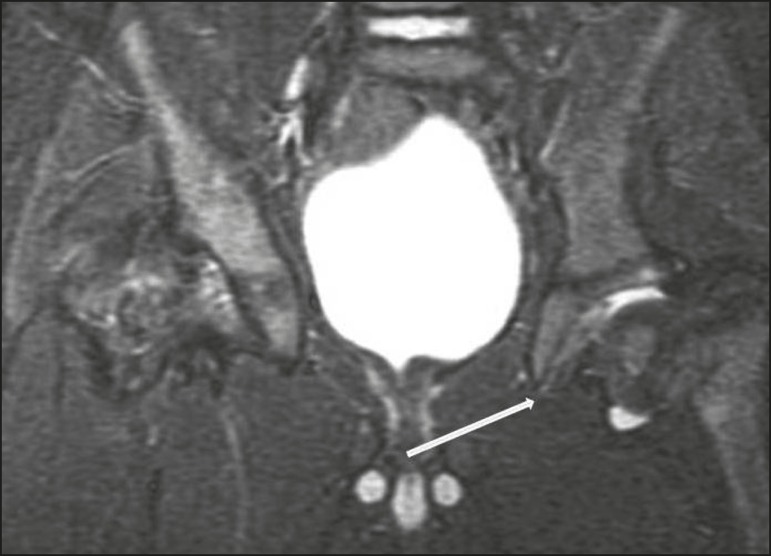
MRI of the hip. STIR coronal sequence showing signs of pronounced epiphysiolysis. Note the moderate joint effusion.

On MRI, epiphysiolysis presents as focal or diffuse physeal enlargement, best defined in coronal or axial T1-weighted sequences, together with irregularity and a hyperintense signal caused by bone marrow edema (Figure 8). 

**Figure 9:**
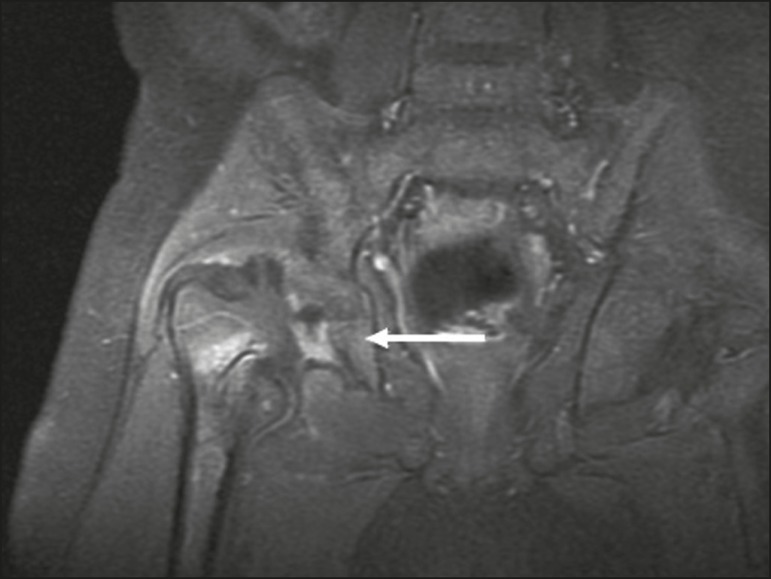
MRI of the hip. Contrast-enhanced, coronal, fat-saturated T1-weighted sequence showing complete destruction/resorption of the femoral head, accompanied by severe deformity of the femoral neck and acetabulum.

Avascular necrosis of the femoral head occurs in approximately 15% of patients with epiphysiolysis. The alterations caused by such necrosis include the crescent sign, juxta-articular sclerosis, cystic areas, flattening, fragmentation, and collapse of the femoral head. Necrosis typically involves the anterolateral femoral head^(13)^. Late complications include pistol-grip deformity (enlargement and shortening of the femoral neck and varus deformity), degenerative osteoarthritis, and limb-length discrepancy resulting from physeal fusion (Figure 9).

MRI is useful in diagnosing pre-epiphysiolysis conditions, when there is no objective sign of femoral head displacement. It is recommended that both hips be evaluated, because epiphysiolysis is bilateral in up to 50% of cases^(13)^.

#### Apophysitis/osteochondritis

Sites of tendon attachment (apophyses) are sensitive to repetitive traction. The apophyses are subject to traction apophysitis or osteochondritis at the insertion site^(13)^. Either can compromise the development of ossification nuclei of the apophyses, with fragmentation and an increase in volume^(13)^. The pelvic sites most commonly involved are the iliac crest, anterosuperior iliac spine, the anteroinferior iliac spine, and the ischial tuberosity, as well as the lesser and greater trochanters^(14)^.

**Figure 10:**
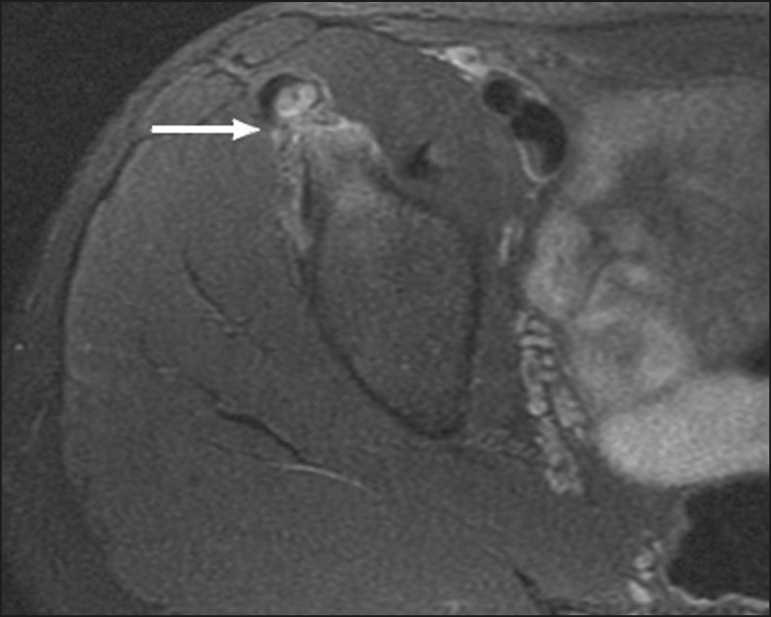
A 14-year-old male. Axial proton density-weighted fat-saturated MRI showing bone edema in the right anteroinferior iliac spine.

In the acute phase of apophysitis, X-rays are useful for confirming the presence of incipient apophysitis, documenting the avulsion or fragmentation of a given apophysis, and ruling out other bone abnormalities such as epiphyseal fractures^(15)^. In patients with apophysitis and no avulsion, X-rays appear normal. Although the MRI findings of apophysitis can be variable, the initial findings include low signal intensity on T1-weighted sequences with increased signal intensity in fluid-sequences, bone marrow edema, and mild enlargement of the physis (Figure 10). After healing, there can be hypertrophic ossification and residual sclerosis^(14)^.

## CONCLUSION

The differential diagnoses of hip pain in the pediatric population are myriad. Therefore, timely, accurate diagnosis is essential to ensure early, effective treatment.

In pediatric patients with hip pain, a measured approach to diagnostic imaging, contextualized by clinical and laboratory data, helps identify the correct diagnosis, avoid unnecessary interventions, properly manage cases, and prevent complications in adulthood.
